# Prediction of protein-RNA residue-base contacts using two-dimensional conditional random field with the lasso

**DOI:** 10.1186/1752-0509-7-S2-S15

**Published:** 2013-12-17

**Authors:** Morihiro Hayashida, Mayumi Kamada, Jiangning Song, Tatsuya Akutsu

**Affiliations:** 1 Bioinformatics Center, Institute for Chemical Research, Kyoto University, Gokasho, Uji, Kyoto 611-0011, Japan; 2 Department of Biochemistry and Molecular Biology, Monash University, Clayton, VIC 3800, Australia; 3 Tianjin Institute of Industrial Biotechnology, Chinese Academy of Sciences, Tianjin 300308, China

## Abstract

**Background:**

To uncover molecular functions and networks in biological cellular systems, it is important to dissect interactions between proteins and RNAs. Many studies have been performed to investigate and analyze interactions between protein amino acid residues and RNA bases. In terms of interactions between residues in proteins, it is generally accepted that an amino acid residue at interacting sites has coevolved together with the partner residue in order to keep the interaction between residues in proteins. Based on this hypothesis, in our previous study to identify residue-residue contact pairs in interacting proteins, we made calculations of mutual information (*M I*) between amino acid residues from some multiple sequence alignment of homologous proteins, and combined it with a discriminative random field (DRF) approach, which is a special type of conditional random fields (CRFs) and has been proved useful for the purpose of extracting distinguishing areas from a photograph in the image processing field. Recently, the evolutionary correlation of interactions between residues and DNA bases has also been found in certain transcription factors and the DNA-binding sites.

**Results:**

In this paper, we employ more generic two-dimensional CRFs than such DRFs to predict interactions between protein amino acid residues and RNA bases. In addition, we introduce labels representing kinds of amino acids and bases as local features of a CRF. Furthermore, we examine the utility of *L*_1_-norm regularization (lasso) for the CRF. For evaluation of our method, we use residue-base interactions between several Pfam domains and Rfam entries, conduct cross-validation, and calculate the average AUC (Area under ROC Curve) score. The results suggest that our CRF-based method using mutual information and labels with the lasso is useful for further improving the performance, especially provided that the features of CRF are successfully reduced by the lasso approach.

**Conclusions:**

We propose simple and generic two-dimensional CRF models using labels and mutual information with the lasso. Use of the CRF-based method in combination with the lasso is particularly useful for predicting the residue-base contacts in protein-RNA interactions.

## Introduction

It is essential to understand the organization and evolution of cellular systems and molecular networks through the analysis of interactions and molecular recognition. Protein-RNA interactions are related with regulatory mechanisms including RNA splicing, post-transcriptional control, protein translation, and so on. Many researchers have focused on tertiary structures of complexes consisting of specific proteins and RNAs, and have analyzed how proteins selectively make physical contacts with specific sites on nucleic acids [1,2]. Some degree of mutual accommodation between the protein binding surfaces and RNA causes the formulation of most protein-RNA complexes. Markus et al. reported that a loop of the L11 RNA binding domain becomes ordered on binding although the loop is absolutely unstructured without the partner RNA [3]. Scherly et al. reported that the same RNA subsequence containing seven bases, AUUGCAC, is recognized by the U1A protein, a part of ribosomes, under the context of an internal loop or hairpin loop [4]. Jones et al. reported that van der Waals contacts are more widely used rather than hydrogen bond contacts in protein-single(double)-stranded DNA and protein-RNA complexes. They pointed out that proteins are likely to use van der Waals contacts and hydrogen bonds in interactions to the pyrimidine uracil and the purine guanine, and prefer phenylalanine, arginine, tyrosine residues in the RNA binding site [2]. Thus, in this paper, we focus on prediction of such residue-base contacts in interacting protein-RNA pairs.

In our previous study, we proposed a prediction method for protein residue-residue contacts [5]. In order to uncover details of interactions between protein amino acid residues, several investigations have been done [6-9]. It is generally accepted that interacting residues in a protein have a pressure to be simultaneously mutated with each other through evolutionary processes to keep their interactions. Under the selection pressure, otherwise, mutations at such interacting sites might lead to loss of the interactions and disappearance of individual. Thus, interacting residues are required to be mutated in a coordinated manner in order to maintain their interactions. Since mutual information (*M I*) is defined as a quantity representing dependent relationship between two random variables, *M I *between positions in a protein, which is obtained from the distribution of amino acids in multiple sequence alignments for its homologous proteins, is useful for predicting interacting residues.

For interactions between protein amino acid residues and DNA bases, Yang et al. showed that the evolutions of the transcription factors and the DNA binding sites of the basic helix-loop-helix family, homeo family, high-mobility group family, and transient receptor potential channels family are significantly correlated across eukaryotes [10]. Accordingly, a mutual information-based method was developed for identifying coevolved protein residues and DNA bases. From analogy to interactions between residues, and between residues and DNA bases, it can be concluded that interacting residues and RNA bases tend to be simultaneously mutated. We therefore utilize *M I *for prediction of residue-base contacts between proteins and RNAs.

Some researchers have developed methods to predict RNA-binding regions in protein sequences. Kumar et al. proposed utilization of evolutionary information and position-specific scoring matrix (PSSM) profiles that PSI-BLAST generates, and predicted using support vector machine (SVM) approach [11]. Furthermore, they developed different hybrid approaches, and improved the prediction accuracy [12]. Kim et al. introduced some propensity in the RNA interface of a protein to measure residue pairing preferences by computationally analyzing tertiary structures of protein-RNA complexes [13]. Muppirala et al. developed a prediction method from only sequence information for interactions between RNAs and proteins, called RPISeq [14]. Liu et al. proposed a novel interaction propensity representing a binding selectivity of a residue to the interacting RNA nucleotide by considering its two-side neighborhood in a residue triplet with combination of other sequence, features based on structures, and the random forest technique [15]. These methods, however, do not predict contacts between specific bases and residues in RNAs and proteins, and only detect RNA-binding regions in proteins.

Markov random fields (MRFs) have been widely used in fields of pattern recognition, image processing, and so on. For modeling of spatial interactions in images, Kumar and Hebert proposed the discriminative random field (DRF) that is defined as a special type of conditional random fields (CRFs), and applied their method to detection of regions of non-natural, artificial buildings from photographs [16]. They maintained that their DRFs have some advantages in comparison with general MRFs. For instance, DRFs are able to discriminate in higher accuracies than MRFs, and can be constructed without the assumption of conditional independence for observed data. It should be noted that such DRFs might not represent actual structures. MRFs and CRFs have been also used in the field of computational biology. Deng et al. proposed an MRF-based method to predict protein functions from protein-protein interaction networks [17,18]. Hayashida et al. proposed a CRF-based method to predict protein-protein interactions using protein domain information [19]. Kamada et al. proposed a DRF approach to predict protein residue contacts [5]. On the other hand, the DRF proposed by Kumar and Hebert [16] is strongly associated with images, and the interaction potential works to smooth borders of regions. Thus, DRFs may not be directly applicable to prediction of protein residue contacts. Hence, instead of DRFs, we propose simple and generic two-dimensional CRF models that accept more interaction structures. In our previous study, we provided ordinary mutual information between two positions obtained from multiple alignments as an input to CRFs [20]. Dunn et al. proposed an improvement of *M I*, called *M I_p_*, and claimed that it dramatically improved residue contact prediction [21]. We therefore examine *M I_p _*as well as *M I*. In addition, we introduce labels representing kinds of amino acids and bases as local features of our CRF models. However, inclusion of more parameters in CRF models may cause overfitting. Hence, we examine *L*_1_-norm regularization, or the least absolute shrinkage and selection operator (lasso) [22] for the purpose of avoidance of overfitting. We perform computational experiments, and the results suggest that the CRF-based method using mutual information and labels with the lasso is useful.

## Method

We propose a prediction method based on simple and generic conditional random fields (CRFs) with *L*_1_-norm regularization (lasso) for amino acid residue-base contacts between RNAs and proteins. It takes the amino acid sequence of a protein and the base sequence of an RNA as input data. Then, a sufficient number of homologous sequences for each sequence is gathered in some adequate manner, and mutual information between a position of the protein and one of the RNA is computed. Our method estimates the probability that the residue at a position and the base at another position interact with each other according to our probability formulation of CRFs. To determine parameters of the CRF model for training data, the method takes several protein-RNA pairs with their sequences, and known pairs of positions that a residue and a base interact.

### Mutual information

**Figure 1:**
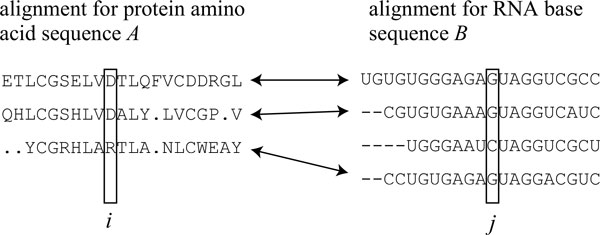
**Illustration on calculation of mutual information**. Illustration on calculation of mutual information between positions *i *and *j *in multiple sequence alignments for protein amino acid sequence *A *and RNA base sequence *B*. In this figure, an arrow indicates that sequences connected with each other by the arrow belong to the same organism, and the third sequence in the alignment for RNA *B *is ignored in calculation of mutual information because it does not have a partner protein sequence of the same organism. Sequences *A *and *B *are shown at the first line of multiple sequence alignments, respectively, and gaps inserted by alignment algorithms are deleted with the columns.

In this section, we briefly review mutual information for distributions of amino acids and bases, and one of its improvements, *M I_p_*, proposed by Dunn et al. [21]. Let *A *and *B *be a protein amino acid sequence and an RNA base sequence, respectively. The calculation of mutual information between two positions in two multiple sequence alignments is illustrated as in Figure 1. A sufficient number of homologous sequences for each of sequences *A *and *B *is gathered, and multiple sequence alignments are constructed in some appropriate manner. Then, gaps inserted to sequences *A *and *B *in the construction of alignments are deleted with the columns because the target of our contact prediction is not such gaps, but amino acid residues in protein *A *and bases in RNA *B*. After the deletion, the length of each multiple alignment becomes the same as that of the original sequence. The example in Figure 1 shows such multiple alignments, in which the first sequence in each alignment indicates sequence *A *or *B*. Let Σ*_a _*and Σ*_b _*be the set of twenty distinct amino acids and one character representing a gap, and the set of four distinct bases and one gap character, respectively. Let *P_i _*(*a*) and *P_j _*(*b*) be the observed frequencies of amino acid *a *(∈ Σ*_a_*) at position *i*, that of base *b *(∈ Σ*_b_*) at position *j*, respectively. Let *P_ij _*(*a, b*) be the joint frequency of amino acid *a *(∈ Σ*_a_*) and base *b *(∈ Σ*_b_*) at positions *i *and *j*. These frequencies are divided by the total number of sequences in a multiple alignment. We assume that the sequence containing amino acid *a *and the sequence containing base *b *belong to the same organism for each pair (*a, b*). Hence, each sequence in a multiple alignment must have a corresponding sequence in another alignment (see Figure 1). Then, mutual information *m_ij _*between two positions *i *in protein *A *and *j *in RNA *B *is defined by

(1) $${m_{i}}_{j}\;=\;\underset{a\in \Sigma_{a}}{\sum }\underset{b\in \Sigma_{b}}{\sum }P_{ij}\left(a,b\right)\log\frac{P_{ij}\left(a,b\right)}{P_{i}\left(a\right)P_{j}\left(b\right)}.$$

However, it has been reported that in some cases it is difficult to identify residue-residue contacts in a protein by *M I *and thus the usefulness is limited [21]. Dunn et al. proposed a metric, *M I_p_*, by removing background noise of *M I*. *M I_p _*for residues at positions *i *and *j *in a protein is defined by

(2) $$m_{ij}\;-\;\frac{\left(\frac{1}{N_{p}-1}\sum_{k\neq i}m_{ik}\right)\left(\frac{1}{N_{p}-1}\sum_{k\neq j}m_{jk}\right)}{\frac{2}{N_{p}\left(N_{p}-1\right)}\sum_{i<j}m_{ij}},$$

where *N_p _*indicates the number of amino acid residues in the protein. For our purpose of the prediction of residue-base contacts, *M I_p _*is modified to $m_{ij}^{\left(p\right)}$ for a pair of a residue at position *i *and a base at position *j *as follows:

(3) $$m_{ij}^{\left(p\right)}\;=\;m_{ij}-\frac{\left(\frac{1}{N_{r}}\sum_{k=1}^{N_{r}}m_{ik}\right)\left(\frac{1}{N_{p}}\sum_{k=1}^{N_{p}}m_{kj}\right)}{\frac{1}{N_{p}N_{r}}\sum_{i=1}^{N_{p}}\sum_{j=1}^{N_{r}}m_{ij}},$$

(4) $$=\;m_{ij}-\frac{\sum_{k=1}^{N_{r}}m_{ik}\sum_{k=1}^{N_{p}}m_{kj}}{\sum_{i=1}^{N_{p}}\sum_{j=1}^{N_{r}}m_{ij}},$$

where *N_p _*and *N_r _*are the number of residues in protein *A *and that of bases in RNA *B*, respectively.

### Two-dimensional conditional random field (CRF) for residue-base contact prediction

In this section, we show our simple and generic two-dimensional CRFs for prediction of residue-base contacts.

Lafferty et al. proposed conditional random fields (CRFs) by extending Markov random fields (MRFs) [23]. Let *G*(*V, E*) be a graph that consists of a set of vertices *V *and a set of edges *E*. In these random fields, each vertex *v *(∈ *V *) is related with a random variable *x_v_*. Then, (***x***, ***y***) is a conditional random field if random variables *x_v _*(∈ ***x***) follow the Markov property under observations ***y ***according to the graph *G*. It means that $Pr\left(x_{v}|x_{\left\{v^{\prime }\in V|v^{\prime }\neq v\right\}},y\right)=Pr\left(x_{v}|{x_{N}}_{v},y\right)$, where $N_{v}$ indicates the set of vertices neighboring with the vertex *v *in *G*. This property requires *Pr*(***x*'**|***y***) > 0 for all subsets ***x' ***of random variables ***x***. Thus, CRFs can be represented as

(5) $$Pr\left(x_{v}|x_{n_{v}},y\right)=\frac{1}{Z_{v}}\exp\left\{-U_{v}\left(x,y\right)\right\},$$

where *U_v _*(***x***, ***y***) indicates a potential function with respect to the vertex *v*, and *Z_v _*indicates the normalization constant defined as $\sum_{x_{v}}\exp\left\{-U_{v}\left(x,y\right)\right\}$.

The discriminative random field (DRF) proposed by Kumar and Hebert [16] is a special type of CRFs. In our previous study [5], we applied the DRF to prediction of residue-residue contacts. The potential function *U_v _*(***x***, ***y***) is defined by

(6) $$U_{v}\left(x,y\right)=A\left(x_{v},y\right)+\beta \underset{v^{\prime }\in N_{v}}{\sum }I\left(x_{v},x_{v^{\prime }},y\right),$$

where *β *is a constant, and random variable *x_v _*takes 1 or -1. The association potential *A*(*x_v_, **y***) and

interaction potential *I*(*x_v_*, *x_v'_*, ***y***) are defined by

(7) $$A\left(x_{v},y\right)\;=\;-\log\left(\sigma \left(x_{v}w_{f}^{T}f_{v}\left(y\right)\right)\right),$$

(8) $$I\left(x_{v},x_{v^{\prime }},y\right)\;=\;\alpha x_{v}x_{v^{\prime }}+\left(1-\alpha \right)\left(2\sigma \left(x_{v}x_{v^{\prime }}w_{g}^{T}g_{vv^{\prime }}\left(y\right)\right)-1\right)$$

respectively, where ***w***_*f *_and ***w***_*g *_indicate vectors of parameters, ***f***_*v *_and ***g***_*vv*' _indicate vector-valued functions of mapping ***y ***to feature vectors, *α *(0 *≤ α ≤ *1) is a constant, $\sigma \left(x\right)=\frac{1}{1+e^{-x}}$, and ***w***^*T *^indicates the transpose of ***w***. It has been shown that the DRF is effective to extraction of distinguishing areas from photo images. The association potential *A*(*x_v_, **y***) represents a gain obtained only from *v *and ***y***, and the interaction potential *I*(*x_v_, x_v'_, **y***) represents a gain obtained from some relationship of *v *with *v'*, and works to smooth the truth assignment for random variables ***x ***because adjacent pixels in photographs are likely to have similar colors to each other. The smoothing property, however, is not desired for predicting contacts between protein residues and RNA bases. Hence, we use the following potential for random variables *r_ij _*∈ {0, 1} representing whether or not the residue and the base at positions *i *and *j *interact with each other, that is to say, *r_ij _*= 1 if they interact, otherwise *r_ij _*= 0.

(9) $$U_{ij}\left(r,y\right)=w_{f}^{T}f_{ij}\left(r,y\right)+w_{g}^{T}\underset{\left(k,l\right)\;\in {N_{i}}_{j}}{\sum }g_{ijkl}\left(r,y\right)$$

Here, it should be noted that the first and second terms in the right-hand side are corresponding to the association and interaction potentials in DRF, respectively. In our CRF model, each vertex in graph *G *is associated with a position pair (*i*, *j*), and the parameter set *θ *consists of ***w***_*f *_and ***w***_*g*_.

**Figure 2:**
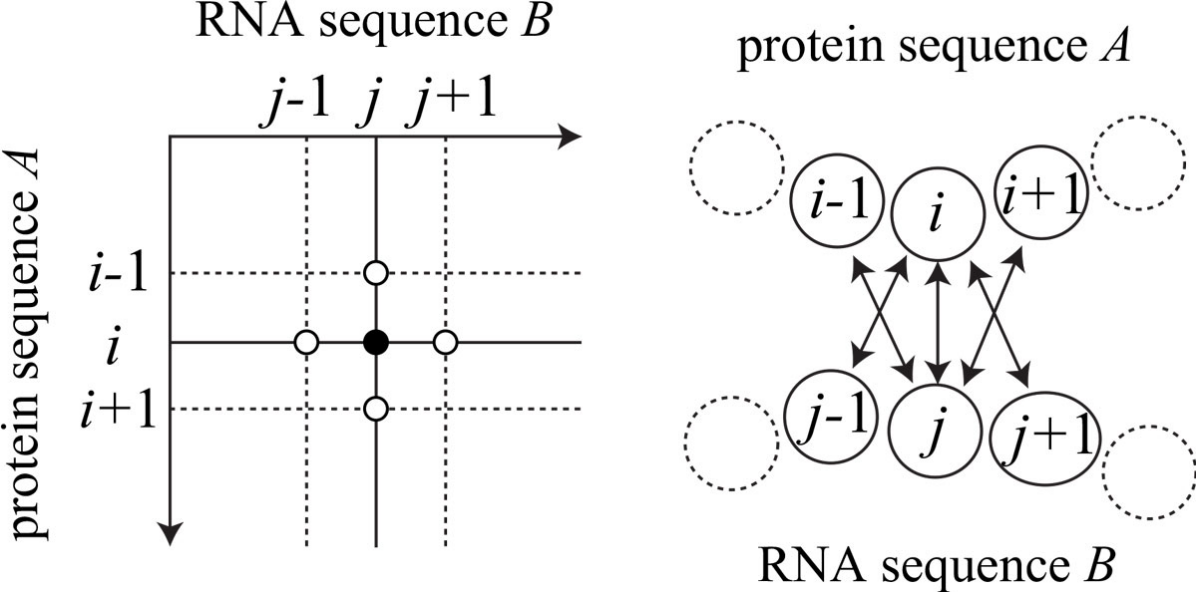
**Neighboring residue-base pairs with (*i, j*) in our two-dimensional conditional random fields**. Neighboring pairs with (*i, j*) are defined as (*i *± 1, *j*), and (*i, j *± 1).

To decide a CRF model, vector-valued functions ***f***_*ij*_, ***g***_*ijkl *_that give local features, and a set $N_{ij}$ of vertices neighboring with vertex (*i*, *j*) must be designed. In this paper, we define neighboring vertices with (*i*, *j*) as $N_{ij}$ = {(*i *± 1, *j*), (*i, j *± 1)} (see Figure 2). In addition, we consider *M I *(*m_ij _*) and *M I_p _*($m_{ij}^{\left(p\right)}$) between positions *i *and *j *as observations ***y***. Then, as a formulation of ***f***_*ij *_and ***g***_*ijkl*_, $f_{ij}^{\left(1\right)}$ and $g_{ijkl}^{\left(1\right)}$ are defined by

(10) $$f_{ij}^{\left(1\right)}\left(r,m\right)\;=\;\left(\begin{array}{c} r_{ij}\\ {\bar{r}}_{ij} \end{array}\right)⊗\left(\begin{array}{c} 1\\ m_{ij} \end{array}\right),$$

(11) $$g_{ijkl}^{\left(1\right)}\left(r,m\right)\;=\;\left(\begin{array}{c} {r_{i}}_{j}\\ {\bar{r}}_{ij} \end{array}\right)⊗\left(\begin{array}{c} r_{kl}\\ {\bar{r}}_{kl} \end{array}\right)⊗\left(\begin{array}{c} 1\\ m_{kl} \end{array}\right),$$

where $\bar{r}$ indicates the negation of *r*, that is, $\bar{1}$ = 0, $\bar{0}$ = 1, and  ⊗ indicates the Kronecker product, for instance, $X⊗Y=\left(\begin{array}{c} x_{1}Y\\ x_{2}Y \end{array}\right)$ for matrices $X=\left(\begin{array}{c} x_{1}\\ x_{2} \end{array}\right)$ and *Y*, and then $f_{ij}^{\left(1\right)}\left(r,m\right)$ can be also written as ${\left(r_{ij},r_{ij}m_{ij},{\bar{r}}_{ij},{\bar{r}}_{ij}m_{ij}\right)}^{T}$.

**Figure 3:**
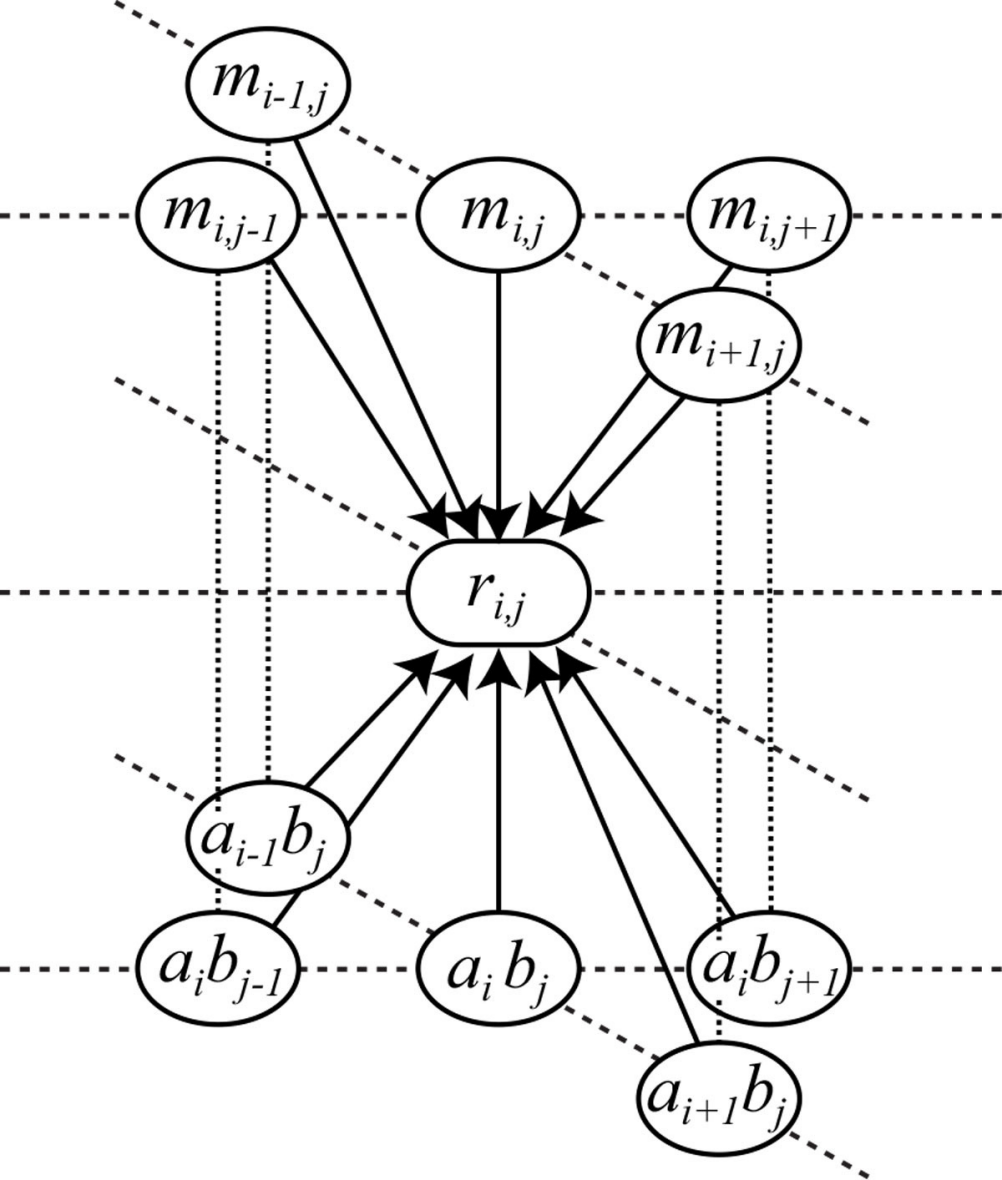
**Relationship between the random variable *r_ij _*and observations in our CRF model**. Relationship between the random variable *r_ij _*and the observations of mutual information *m_ij_*, and the pair (*a_i_, b_j _*) of the *i*-th amino acid in protein sequence *A *and the *j*-th base in RNA sequence *B*, in our CRF model.

In addition to mutual information, we introduce labels representing kinds of amino acids and bases in the target protein and RNA sequences as observations. Suppose protein sequence *A *and RNA sequence *B *are represented by *a*_1_*a*_2 _⋯ *a_Np _*and *b*_1_*b*_2 _⋯ *b_Nr_*, respectively. Then, As another formulation, $f_{ij}^{\left(2\right)}$ and $g_{ijkl}^{\left(2\right)}$ are defined by

(12) $$f_{ij}^{\left(2\right)}\left(r,m,a,b\right)\;=\;\left(\begin{array}{c} r_{ij}\\ {\bar{r}}_{ij} \end{array}\right)⊗\delta_{\left(a_{i},\;b_{j}\right)}⊗\left(\begin{array}{c} 1\\ m_{ij} \end{array}\right),$$

(13) $$g_{ijkl}^{\left(2\right)}\left(r,m,a,b\right)\;=\;\left(\begin{array}{c} r_{ij}\\ {\bar{r}}_{ij} \end{array}\right)⊗\left(\begin{array}{c} r_{kl}\\ {\bar{r}}_{kl} \end{array}\right)⊗\delta_{\left(a_{k},b_{l}\right)}⊗\left(\begin{array}{c} 1\\ m_{kl} \end{array}\right),$$

respectively, where *δ *_(*a, b*) _(*a *∈ Σ*_A_, b *∈ Σ*_B_*) without grouping amino acids indicates a 0-1 constant vector having size 20 × 4 = 80 that the element corresponding to (*a*, *b*) is 1 and the others are 0. Figure 3 shows the relationship of the random variable *r_ij _*at sequence positions (*i*, *j*) with observations including mutual information *m_ij_*, amino acids *a_i_*, and bases *b_j_*, in our CRF model. It means that *r_ij _*is related with observations *m_ij _*and (*a_i_*, *b_j _*) at multiple neighboring positions, which is an important property of CRFs different from MRFs. Besides, we consider another model witout mutual information for the purpose of model comparison as follows:

(14) $$f_{ij}^{\left(3\right)}\left(r,a,b\right)\;=\;\left(\begin{array}{c} r_{ij}\\ {\bar{r}}_{ij} \end{array}\right)⊗\delta_{\left(a_{i},b_{j}\right)},$$

(15) $$g_{ijkl}^{\left(3\right)}\left(r,a,b\right)\;=\;\left(\begin{array}{c} r_{ij}\\ {\bar{r}}_{ij} \end{array}\right)⊗\left(\begin{array}{c} r_{kl}\\ {\bar{r}}_{kl} \end{array}\right)⊗\delta_{\left(a_{k},b_{l}\right)}.$$

### Estimation of parameters in two-dimensional CRFs

We can estimate parameters *θ *= {***w***_*f*_, ***w***_*g*_} from training data by maximizing a pseudo-likelihood function as described in [5,16]. Let *N *be the number of pairs of given protein and RNA sequences. Let ***a***^(*n*) ^and ***b***^(*n*)^(*n *= 1, ..., *N*) be the *n*-th protein and RNA sequences, respectively. Let ***r***^(*n*) ^be the residue-base contacts for the *n*-th protein-RNA pair. Then, *M I *(and also *M I_p_*) ***m***^(*n*) ^is calculated for the *n*-th pair. The logarithm pseudo-likelihood function *L*(*θ*) is defined by

(16) $$L\left(\theta \right)=\log\overset{N}{\underset{n=1}{\prod }}\underset{i}{\prod }\underset{j}{\prod }Pr\left(r_{ij}^{\left(n\right)}|r_{Nij}^{\left(n\right)},m^{\left(n\right)},a^{\left(n\right)},b^{\left(n\right)},\theta \right)$$

We employ the Broyden-Fletcher-Goldfarb-Shanno (BFGS) method [24] to find parameters *θ *maximizing *L*(*θ*), which is a quasi-Newton method that approximates the Hessian matrix by some efficient method using partial differentials. For our problem, the following formulae of *L*(*θ*) partially differentiated by each parameter vector ***w ***(∈ {***w***_*f*_, ***w***_*g*_}) are required.

(17) $$\begin{array}{c} \frac{\partial L\left(\theta \right)}{\partial w}=\underset{n}{\sum }\underset{i}{\sum }\underset{j}{\sum }\left\{-\frac{\partial U_{ij}\left(r^{\left(n\right)},m^{\left(n\right)},a^{\left(n\right)},b^{\left(n\right)},\theta \right)}{\partial w}\right)\\ \;\;\left(+\underset{r_{ij}^{\left(n\right)}}{\sum }Pr\left(r_{ij}^{\left(n\right)}|r_{N_{ij}}^{\left(n\right)},m^{\left(n\right)},a^{\left(n\right)},b^{\left(n\right)},\theta \right)\frac{\partial U_{ij}\left(r^{\left(n\right)},m^{\left(n\right)},a^{\left(n\right)},b^{\left(n\right)},\theta \right)}{\partial w}\right\}, \end{array}$$

where

(18) $$\frac{\partial U_{ij}\left(r^{\left(n\right)},m^{\left(n\right)},a^{\left(n\right)},b^{\left(n\right)},\theta \right)}{\partial w_{f}}=f_{ij}\left(r^{\left(n\right)},m^{\left(n\right)},a^{\left(n\right)},b^{\left(n\right)}\right),$$

(19) $$\frac{\partial U_{ij}\left(r^{\left(n\right)},m^{\left(n\right)},a^{\left(n\right)},b^{\left(n\right)},\theta \right)}{\partial w_{g}}=\underset{\left(k,l\right)\in N_{ij}}{\sum }g_{ijkl}\left(r^{\left(n\right)},m^{\left(n\right)},a^{\left(n\right)},b^{\left(n\right)}\right).$$

It should be noted that parameters *θ *to be estimated are not included in $\frac{\partial U_{ij}}{\partial w}$.

In addition, we propose to use *L*_1_-norm regularization, or the least absolute shrinkage and selection operator (lasso) [22]. That is, we maximize the following function.

(20) $$L\left(\theta \right)-C\left(||w_{f}|{|}_{1}+||w_{g}|{|}_{1}\right),$$

where *C *is a positive constant, and *||**w**||*_1 _indicates *L*_1 _norm of ***w***, $\sum_{i=1}^{n}|w_{i}|$ for ***w ***= (*w*_1_, ⋯, *w_n_*)*^T^*.

### Contact inference

We determine whether or not a new residue-base pair forms a contact depending on the CRF with the parameters estimated by the method described in the previous section. Although we used the iterated conditional modes (ICM) [25] in our previous study, it has been recognized that ICM often converges to local solutions in image processing benchmark problems [26]. In this paper, therefore, we apply an improved algorithm of the tree-reweighted message passing (TRW) algorithm [27], the sequential tree-reweighted message passing (TRW-S) algorithm [28]. These method iteratively update messages *M*_*vv'*;*x *_from a vertex *v *to another *v' *with state *x*, and iteratively replace edge weights ***w ***for all trees decomposed from the original graph, to minimize the upper bound of the objective function for a maximization problem. In our two-dimensional CRF model, the vertex *v *and the state *x *mean a position pair (*i, j*) and a random variable *r_ij_*, respectively, and then *v' *∈ $N_{ij}$.

## Computational experiments

### Data and implementation

**Figure 4:**
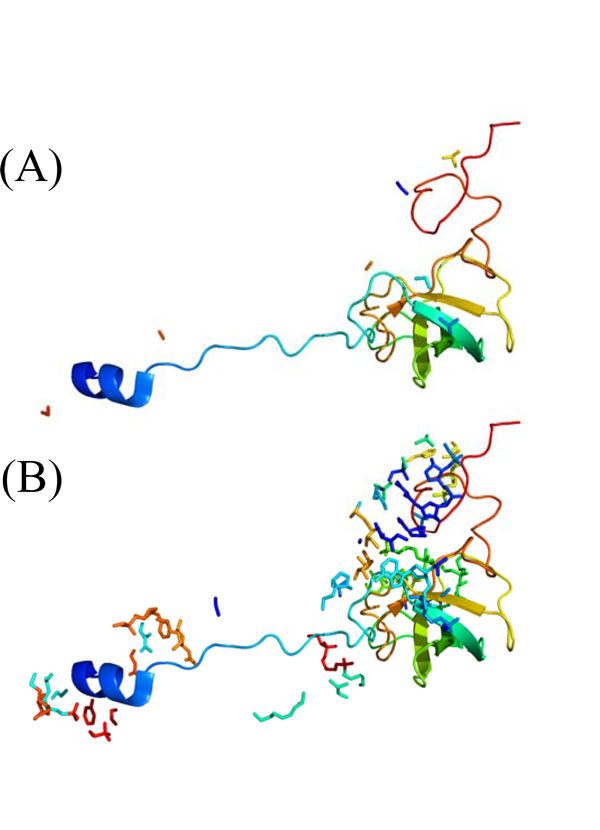
**Example of residue-base contacts**. (A) Protein RS12_THET8, chain 'O' of PDB code '1yl4', and the atoms of RNA M26923, chain 'A' within 3 Å of the protein. (B) Protein RS12_THET8 and the atoms of RNA M26923 within 5 Å of the protein. It should be noted that for the RNA molecule, only atoms within 3 Å/5Å of the protein are shown.

For the evaluation of our method, we used tertiary structures of protein-RNA complexes in the PDB databank [29], and prepared thirteen protein-RNA pairs, (RL18_THETH, X01554), (RL27_ECOLI, J01695), (RL27_THET8, X12612), (RL33_THET8, X12612), (RL35_ECOLI, J01695), (RS5_ECOLI, J01695), (RS7_ECOLI, J01695), (RS8 _HET8, M26923), (RS10_THET8, M26923), (RS12_THET8, M26923), (RS15_ECO57, J01695), (RS17_ECOLI, J01695), and (RS17_THET8, M26923), which are contained in ribosomes, '1yl4', '2hgu', '3kc4' and '3kcr' in PDB code. It should be noted that to get contacts between residues and bases, the sequences stored in PDB for these proteins and RNAs must be the same as those included in multiple sequence alignments of the corresponding Pfam [30] and Rfam [31] entries, respectively, and the sequence in a PDB entry is not always the same as that in UniProt [32] entry referred from the PDB entry. We used only the PDB entries in which the sequence is the same as that in UniProt. For each protein-RNA pair of the dataset, Table 1 shows the followings: the identifiers of UniProt, Pfam, and the chain in PDB, the length of protein sequence A, the identifiers of GenBank [33], Rfam, and the chain, the length of RNA sequence B, the PDB code, the number of sequences in the multiple alignment combined on the basis of the organisms, and the number of contacts within 3 Å and that within 5Å. We supposed that a residue and a base form a contact if the Euclidean distance between an atom of the residue and one of the base is less than or equal to some threshold. In this paper, we examined 3 Å and 5 Å as the threshold of contacts because the distances of hydrogen bonds between oxygen and nitrogen atoms, OH-O, OH-N, NH-O, and NH-N, are about 2.7 to 2.9 Å. For instance, protein RS12_THET8 (chain 'O' of '1yl4') and the atoms of RNA_M26923 (chain 'A') within 3 Å of the protein are shown in Figure 4A, and on the other hand, the protein and the atoms of the RNA within 5 Å of the protein is shown in Figure 4B.

**Table 1:** Dataset of thirteen interacting protein-RNA pairs

protein sequence A				RNA sequence B				PDB code	# sequences in MSA	# contacts	
			
UniProt	Pfam	chain	length	GenBank	Rfam	chain	length			*≤ *3 Å	*≤ *5 Å
RL18_THETH	PF00861	R	110	X01554	RF00001	B	117	2hgu	1543	28	85
RL27_THET8	PF01016	Z	81	X12612	RF01118	A	108	2hgu	1356	20	67
RL27_ECOLI	PF01016	W	77	J01695	RF01118	8	108	3kcr	1356	18	69
RL33_THET8	PF00471	5	48	X12612	RF01118	A	108	2hgu	1445	18	40
RL35_ECOLI	PF01632	3	61	J01695	RF01118	8	108	3kcr	1337	12	38
RS5_ECOLI	PF00333	E	67	J01695	RF00177	A	1530	3kc4	1701	13	57
RS7_ECOLI	PF00177	G	147	J01695	RF00177	A	1530	3kc4	1941	25	127
RS8_THET8	PF00410	K	135	M26923	RF00177	A	1515	1yl4	1889	29	93
RS10_THET8	PF00338	M	97	M26923	RF00177	A	1515	1yl4	1711	20	84
RS12_THET8	PF00164	O	122	M26923	RF00177	A	1515	1yl4	1972	45	161
RS15_ECO57	PF00312	O	83	J01695	RF00177	A	1530	3kc4	1821	21	89
RS17_ECOLI	PF00366	Q	69	J01695	RF00177	A	1530	3kc4	1690	18	85
RS17_THET8	PF00366	T	69	M26923	RF00177	A	1515	1yl4	1690	29	93

For each protein-RNA pair, the identifiers of UniProt, Pfam, and the chain in PDB, the length of protein sequence A, the identifiers of GenBank, Rfam, and the chain, the length of RNA sequence B, the PDB code, the number of sequences in the multiple sequence alignment (MSA) combined on the basis of the organisms, and the number of contacts within 3 Å and that within 5 Å are shown.

In order to calculate *M I *and *M I_p_*, we used the file 'Pfam-A.full' of Pfam database (release 26.0) [30] and 'Rfam.full' of Rfam database (release 10.1) [31] for getting multiple sequence alignment data of proteins and RNAs, respectively. In counting the frequencies of amino acids and bases, we also examined several classifications of amino acids with 2, 4, 8, 10, and 15 groups proposed by Murphy et al. [34] as shown in Table 2.

**Table 2:** Classification of amino acids

# groups	classification of amino acids
2	MLVICGATSPFYW/DENQRKH
4	MLVIC/GATSP/FYW/DENQRKH
8	MLVIC/GA/TS/P/FYW/DENQ/RK/H
10	MLVI/C/G/A/TS/P/FYW/DENQ/RK/H
15	MLVI/C/G/A/T/S/P/FY/W/D/E/N/Q/RK/H

Classification of amino acids by Murphy et al. [34]. The two groups are classified by the hydrophobic and hydrophilic properties of amino acid side-chains. The group of (FYW) is aromatic hydrophobic, (TS), (DENQ), and (RK) are polar.

For the parameter estimation of our CRF, as an implementation of BFGS methods, libLBFGS (version 1.10), available from http://www.chokkan.org/software/liblbfgs/, was used with default options, which carries out the limited memory BFGS method [35]. For the contact inference, as an implementation of the TRW-S method [28], MRF energy minimization software (version 2.1), available from http://vision.middlebury.edu/MRF/code/, was modified for use depending on our pseudo-likelihood function formulation.

## Results

For the evaluation of our proposed CRF, computational experiments were performed in both contact definitions of 3 Å and 5 Å. Three types of local features $\left\{f_{ij}^{\left(1\right)},g_{ijkl}^{\left(1\right)}\right\}$, $\left\{f_{ij}^{\left(2\right)},g_{ijkl}^{\left(2\right)}\right\}$, and $\left\{f_{ij}^{\left(3\right)},g_{ijkl}^{\left(3\right)}\right\}$, five types of grouping amino acids as 2, 4, 8, 10, and 15 groups [34] as shown in Table 2, and lasso parameter *C *= 0, 1, and 2 were examined. We performed cross-validation procedures, in which each procedure used all residue-base pairs contained in one protein-RNA pair of the dataset for test, and those in the other protein-RNA pairs for training. The conditional probability *Pr*(*r_ij _*= 1|$r_{N_{ij}}$, ***m**, **a**, **b**, θ*) and the average AUC (Area Under ROC Curve) score were calculated.

**Figure 5:**
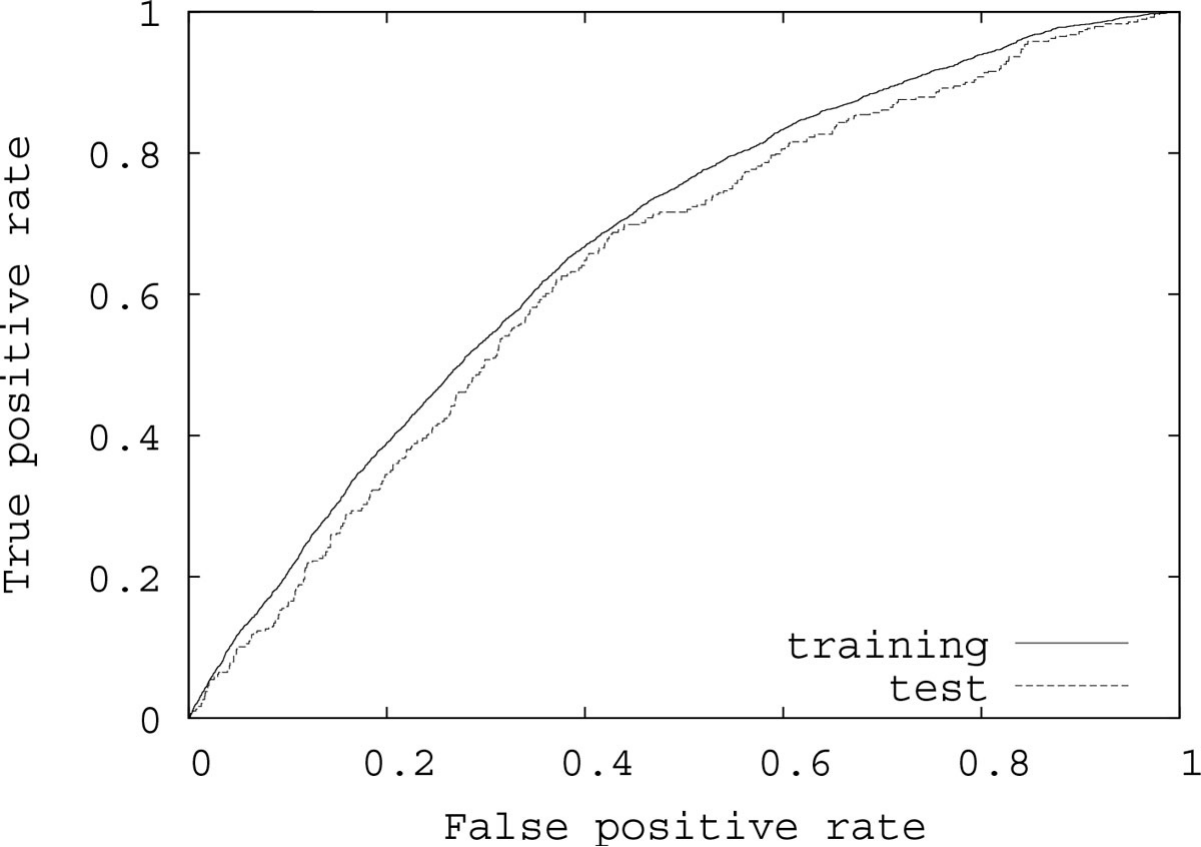
**Average ROC curves of the best case in our experiments for training and test pairs**. Average ROC curves for training and test pairs using both of *M I_p _*and labels with the classification of 15 groups with lasso parameter *C *= 2 using the contact definition of 3 Å.

Tables 3 and 4 show results on the average AUC scores for test protein-RNA pairs using the contact definitions of 3 Å and 5 Å, respectively, under several conditions. '*M I*' ('*M I_p_*') indicates the CRF model having only features of *M I *(*M I_p_*), that is, the feature vectors are $\left\{f_{ij}^{\left(1\right)},g_{ijkl}^{\left(1\right)}\right\}$, '*M I *+ label' ('*M I_p _*+ label') indicates the model having *M I *(*M I_p_*) and labels representing kinds of bases and classified amino acids, $\left\{f_{ij}^{\left(2\right)},g_{ijkl}^{\left(2\right)}\right\}$, and 'label' indicates the model having only labels, $\left\{f_{ij}^{\left(3\right)},g_{ijkl}^{\left(3\right)}\right\}$. It should be noted that the same grouping of amino acids was used in the calculation of *M I *and *M I_p _*and in the labels of features for each case of our experiments. The average AUC score using both of the improved mutual information and labels '*M I_p_*+label' with the grouping of 15 groups with lasso parameter *C *= 2 using the contact definition of 3 Å was best for the tested residue-base pairs. Figure 5 shows the average ROC (Receiver Operating Characteristic) curves for training and test pairs in that case, where the average AUC score for training pairs was 0.673. In many cases, the average AUC scores of '*M I_p_*' were better than those of '*M I*'. It suggests that *M I_p _*is useful also for prediction of residue-base contacts. However, the AUC scores of '*M I_p_*+ label' were comparable with those of '*M I*+label'. It is considered because in $f_{ij}^{\left(2\right)}$ and $g_{ijkl}^{\left(2\right)}$ features of labels largely affected the results. On the other hand, for the CRF models having features of labels, the AUC scores with the lasso were better than those without the lasso in most cases. It means that the lasso was able to reduce the dimension of parameters concerning labels well. However, the reduction using the contact definition of 5 Å was smaller than that using the contact definition of 3 Å. This might be that false positives increase with the relaxation of contact definitions, which restricted the reduction by the lasso. In such a case, it may be necessary to prepare interacting residue-base pairs manually.

**Table 3:** Results on average AUC scores for test pairs using the contact definition of 3 Å

# groups	*M I*	*M I_p_*	label	*M I*+label	*M I_p_*+label
without lasso (*C *= 0)					

2	0.550	0.557	0.503	0.511	0.502
4	0.534	0.517	0.547	0.505	0.502
8	0.541	0.555	0.535	0.512	0.521
10	0.528	0.557	0.519	0.529	0.536
15	0.538	**0.579**	0.533	0.498	0.523
20	0.539	0.574	0.546	0.561	0.557

lasso (*C *= 1)					

2	0.556	0.570	0.505	0.520	0.492
4	0.525	0.542	0.611	0.615	0.596
8	0.509	0.562	0.610	0.603	0.600
10	0.525	0.553	0.634	0.633	0.629
15	0.510	0.569	**0.635**	0.634	0.621
20	0.510	0.579	0.625	0.631	0.622

lasso (*C *= 2)					

2	0.533	0.521	0.510	0.504	0.508
4	0.533	0.543	0.620	0.623	0.620
8	0.550	0.529	0.632	0.624	0.618
10	0.525	0.527	0.625	0.628	0.633
15	0.516	0.524	0.640	0.640	**0.645**
20	0.514	0.546	0.626	0.641	0.642

Results on average AUC scores for test pairs using the contact definition of 3 Å, *M I*, *M I_p_*, labels representing kinds of amino acids and bases, and the grouping of amino acids with lasso parameter *C *= 0, 1, and 2.

**Table 4:** Results on average AUC scores for test pairs using the contact definition of 5 Å

# groups	*M I*	*M I_p_*	label	*M I*+label	*M I_p_*+label
without lasso (*C *= 0)					

2	0.550	0.520	0.568	0.547	0.565
4	0.543	0.506	0.584	0.563	0.581
8	0.541	0.576	0.584	0.578	0.570
10	0.527	**0.588**	0.545	0.528	0.560
15	0.527	0.587	0.539	0.526	0.518
20	0.530	0.570	0.539	0.506	0.508

lasso (*C *= 1)					

2	0.527	0.570	0.564	0.575	0.562
4	0.552	0.555	0.582	0.571	0.575
8	0.510	0.559	0.581	0.584	0.590
10	0.511	0.567	0.587	0.579	0.590
15	0.523	0.571	0.571	0.578	0.574
20	0.514	0.572	0.581	0.587	**0.592**

lasso (*C *= 2)					

2	0.543	0.585	0.581	0.567	0.566
4	0.513	0.557	0.582	0.584	0.580
8	0.509	0.568	0.576	0.574	0.579
10	0.500	0.563	0.594	0.588	0.590
15	0.505	0.591	0.583	0.576	0.582
20	0.502	0.566	0.594	0.598	**0.602**

Results on average AUC scores for test pairs using the contact definition of 5 Å, *M I*, *M I_p_*, labels representing kinds of amino acids and bases, and the grouping of amino acids with lasso parameter *C *= 0, 1, and 2.

Table 5 shows results on average elapsed time (sec) for an iteration of the cross validation using the contact definition of 3 Å, *M I_p_*, labels representing kinds of amino acids and bases, and the grouping of amino acids with lasso parameter *C *= 0, 1, and 2. It should be noted that in an iteration, about 1140000 residue-base pairs on average were used as training data for parameter estimation and about 95000 residue-base pairs were used as test data. Each computational experiment was conducted using a Xeon CPU 3.47GHz. The average elapsed times by '*M I_p_*+label' were longer than those by '*M I_p_*' and 'label' because '*M I_p_*+label' uses more parameters. For the methods using labels, the average elapsed times with the lasso were shorter than those without the lasso in most cases. It means that parameter reduction by the lasso contributed to the decrease of execution time. All together, these results suggest that the CRF-based method using mutual information and labels representing kinds of amino acids and bases with the lasso is very useful for further improving the prediction performance.

**Table 5:** Results on average elapsed time

# groups	*M I_p_*			label			*M I_p_*+label		
	
	*C *= 0	1	2	*C *= 0	1	2	*C *= 0	1	2
2	55.5	43.5	41.5	46.2	46.9	46.2	80.8	64.6	62.6
4	57.9	51.9	42.8	50.6	47.7	48.0	127.7	63.8	62.5
8	56.9	55.8	55.6	54.3	50.5	50.8	194.9	68.2	67.1
10	54.2	57.4	52.5	57.1	52.2	51.8	235.1	73.0	72.8
15	55.6	57.2	55.2	65.2	55.5	55.1	342.5	79.8	79.2
20	57.8	60.4	55.2	68.1	58.2	58.3	320.8	84.6	82.9

Results on average elapsed time (sec) for an iteration of the cross validation using the contact definition of 3 Å, ***M I***_*p*_, labels representing kinds of amino acids and bases, and the grouping of amino acids with lasso parameter *C *= 0, 1, and 2.

## Conclusion

We addressed residue-base contacts between proteins and RNAs, and developed the conditional random field (CRF)-based prediction method, which used labels representing kinds of classified amino acids and bases as local features of the CRF combined with mutual information. In addition, we applied *L*_1_-norm regularization (lasso) to our CRF-based method for avoiding overfitting. For the evaluation of our proposed method, thirteen protein-RNA pairs included in PDB were used in computational experiments, and the average AUC score for test datasets was calculated. From the results, it is seen that the CRF-based method using mutual information and labels representing kinds of amino acids and bases with the lasso is very useful. Furthermore, our proposed CRFs have another advantage. In the previous study [5], the optimization method to the discriminative random field (DRF) with interaction potentials representing relationships between neighboring vertices did not converge. On the other hand, in this paper, our generic two-dimensional CRFs improved this aspect, and was able to deal with interaction potentials for prediction of residue-base contacts. The problem of predicting residue-base contacts, however, is still difficult, and the prediction accuracy was not satisfying. Hence, high-quality datasets of residue-base contacts may need to be prepared with the assistance of biological experts although in this paper contact data were generated depending on only distances between atoms included in a residue and a base. Besides, we can consider use of other measures representing the correlation of a residue with a base instead of mutual information to further improve our prediction method. Modifying local features and potentials in the CRF is also another future work.

## Competing interests

The authors declare that they have no competing interests.

## Authors' contributions

MH developed and implemented the methods. MH drafted the manuscript. MK, JS and TA participated in the discussions during the development of the methods and helped draft the manuscript. All authors read and approved the final manuscript.

## Declarations

The publication of this article has been funded by Grants-in-Aid #22240009 and #24500361 from MEXT, Japan. This work was also supported by grants from the Hundred Talents Program of the Chinese Academy of Sciences (CAS), the National Natural Science Foundation of China (61202167), the Knowledge Innovation Program of CAS (KSCX2-EW-G-8), Tianjin Municipal Science & Technology Commission (10ZCKFSY05600) and the National Health and Medical Research Council of Australia (NHMRC) (490989). JS is an NHMRC Peter Doherty Fellow, and a Recipient of the Hundred Talents Program of CAS and the Japan Society for the Promotion of Science (JSPS) Short-term Invitation Fellowship to the Bioinformatics Center, Kyoto University, Japan.

This article has been published as part of *BMC Systems Biology *Volume 7 Supplement 2, 2013: Selected articles from The 6^th ^International Conference of Computational Biology. The full contents of the supplement are available online at http://www.biomedcentral.com/bmcsystbiol/supplements/7/S2.
